# Editorial: Community series in identification, function, and mechanisms of interferon induced genes associated with viruses, volume II

**DOI:** 10.3389/fimmu.2023.1269413

**Published:** 2023-08-16

**Authors:** Jieying Bai, Linzhu Ren, Chang Li

**Affiliations:** ^1^ Non-Human Primate Research Center, Institute of Molecular Medicine, Peking University, Beijing, China; ^2^ College of Animal Sciences, Key Lab for Zoonoses Research, Ministry of Education, Jilin University, Changchun, China; ^3^ Research Unit of Key Technologies for Prevention and Control of Virus Zoonoses, Chinese Academy of Medical Sciences, Changchun Veterinary Research Institute, Chinese Academy of Agricultural Sciences, Changchun, China

**Keywords:** interferons, interferon stimulated genes, innate immunity, infection, interaction

Research on the antiviral mechanisms mediated by interferon (IFN) and IFN-stimulated genes (ISGs) is a longstanding topic (1) within the field. The continuous discovery of new IFN or its subtype and ISG is of great significance to clarify the new antiviral mechanisms and the interaction between host and pathogen (1, 2). However, we have a long way to go before we fully understand these IFNs and ISGs. Based on a previous collection of articles (1), this Research Topic continues this work related to IFNs and ISGs.

To explore the stimulation of IFNs during porcine alphaherpesvirus pseudorabies virus (PRV), Yin et al. evaluated the expression of type I and III IFNs and their antiviral activities against PRV in different porcine epithelial cells: porcine kidney epithelial cells (PK-15), primary respiratory epithelial cells (PoREC) and intestinal porcine epithelial cells (IPEC-J2). The results showed that PRV induced a variety of infection-dependent type I IFN responses and a prominent III IFN response in PK-15 cells, whereas a rapid and temporal expression of type I and type III IFNs were triggered in IPEC-J2 cells, and no detectable type I or type III IFN responses were observed in PoREC. Surprisingly, both type I and type III IFNs in the pretreatment group exhibited antiviral activities against PRV, but only IFN-α in PK-15 cells and type III IFN in IPEC-J2 cells could effectively inhibit PRV infection. Moreover, Daza-Cajigal et al. reported that partial JAK1 deficiency impairs STAT1 phosphorylation and IFN-γ-inducible gene expression in THP-1 cells, and IFN-γ-induced phagosome acidification and apoptosis in myeloid cells. Partial JAK1 deficiency also weakened the antiviral response in EBV-B cells but enhances the survival of mycobacterial and salmonella in myeloid cells. These results indicate that the IFN responses induced by viral infection depend on the virus-infected cell types.

Furthermore, the stimulation of IFN response also depends on the virus and its replication ability and adaptability in cells. For example, Laine et al. found that the replication of sublineages Omicron BA.1, BA.2, and recombinant sublineage XJ in human lung epithelial Calu-3 cells was weakened compared to Alpha and Delta. The activation of the primary innate immune signaling pathway by SARS-CoV-2 variants is relatively weak, however, all variants stimulate enough interferon to induce the activation of STAT2 and the production of ISGs.

Another contribution in this Research Topic also describes the induction and activation of IFNs on immune cells and ISGs. Li et al. found that the STING-IKKβ-Relish-AMPs axis acts a critical role in shrimp against Vibrio parahaemolyticus infection. After being induced, type I IFNs can stimulate B cells and classical dendritic cells (cDCs) through Th1 and Tfh cell-dependent pathways, thus driving the formation of the germinal center (GC) and the distribution of the IgG subclass against the pathogens (Dahlgren et al.). The interferon response networks induced by lipopolysaccharide (LPS) can also be used to predict the level of severe lower respiratory infections in infancy (Read et al.). Among the networks, IRF1 is identified as a master regulator of the IFN response. In addition, type I IFNs, especially IFNα14 and IFNβ, exhibit super activation on Natural killer (NK) cells, which can enhance the anti-leukemic function of NK cells and prolong the survival of leukemia mice models (Barnes et al.). The unmodified mRNA vaccine can induce type I IFNs or its downstream signaling cascades, which play crucial roles in inducing robust anti-tumor T-cell response to control tumor growth and metastasis (Sittplangkoon et al.). These results further confirm that IFNs and ISGs not only have antiviral and antibacterial effects but also play important roles in anticancer.

Notably, IFN-based therapy may also increase the risk of autoimmune thyroid diseases in patients with HCV infection (Chou et al.). The upregulated type I IFNs and ISGs can enhance myeloid DC CD1C^+^ subpopulation in patients with mutations in three prime repair DNA exonuclease 1 (TREX1), which may associate with the perpetuation of TREX1-induced chilblain lupus and other type I interferonopathies (Eugster et al.). The question of how to effectively activate IFN responses, what makes it play an effective anti-pathogen and anti-cancer role, and how to reduce or control its side effects are among the problems that need to be solved urgently.

In summary, IFN responses and induced-ISGs are double-edged swords, and how to make effective use of them will be an important research hotspot in the future. This community series further provides strong theoretical support for the application and research of IFNs and ISGs. With further applications of multi-omics and high-throughput technology, research on and applications of IFNs and ISGs will also be vigorously promoted in this field in the future. 

## Author contributions

CL: Conceptualization, Writing – original draft, Writing – review & editing. JB: Conceptualization, Writing – original draft, Writing – review & editing. LR: Conceptualization, Writing – original draft, Writing – review & editing.
